# P243: Evaluation of the management of infectious risk in a neonatal unit in Dakar

**DOI:** 10.1186/2047-2994-2-S1-P243

**Published:** 2013-06-20

**Authors:** A Ndir, A Diop, M Faye, P Astagneau, B Ndoye

**Affiliations:** 1 Pronalin, Ministry of Health, Dakar, Senegal; 2 Hôpital Albert Royer, Dakar, Senegal; 3 CCLIN-Paris Nord, Paris, France; 4 ICAN/RIPAQS, Dakar, Senegal

## Introduction

A surveillance of multiresistant bacteria was conducted from April to October 2012 in 2 pilot hospitals in Dakar. Results revealed higher infection rates caused by extended-spectrum beta-lactamase producing *Enterobacteriaceae* (ESBL) in neonatal unit associated with an alarming mortality rate.

## Objectives

The objective of this study was to evaluate the management of infectious risk in the neonatal unit.

## Methods

An extensive investigation was carried out. Following indicators were evaluated: existence and functionality of the infection control committee (ICC), availability of resources for hand hygiene, knowledge and perception of healthcare workers (HCW) on hand hygiene (with tools elaborated by World Health Organization), invasive procedures care and presence of ESBL in the patient environment (by swabbing).

## Results

The ICC was set up but was not functional (no regular meetings, no infection control activities realised). Alcohol-based handrub and disposable towels were rarely found in the unit. More than 80% of HCW didn’t know the hand hygiene indications and thought that handwashing was more effective against germs than handrubbing. Mechanical ventilation and parental nutrition were used in more than 80% of neonates with ESBL infection. The environmental analysis revealed the presence of ESBL strains on surfaces, beds and antiseptic vials.

## Conclusion

Our investigations indicated the potential factors that could explain the high rates of ESBL infection observed in the neonatal unit. The ICC should be functional because of its central role in the management of infectious risk. Material resources (particularly alcohol-based handrub) should be provided in the unit to ensure good hand hygiene practices. Educational programmes on hand hygiene indications and technics will be implemented for all HCW. Guidelines for the cleaning, disinfection and decontamination must be produced. Moreover, an evaluation of practices should be planned regularly to ensure adequate practices.

## Disclosure of interest

None declared

